# Retract *p* < 0.005 and propose using JASP, instead

**DOI:** 10.12688/f1000research.13389.2

**Published:** 2018-02-16

**Authors:** Jose D. Perezgonzalez, M. Dolores Frías-Navarro

**Affiliations:** 1 Business School, Massey University, Manawatu Campus,P.O.Box 11-222, Palmerston North , 4442, New Zealand; 2 Department of Methodology of the Behavioural Sciences, Faculty of Psychology, University of Valencia, Avenida Blasco Ibañez 21, Valencia, 46010, Spain

**Keywords:** practical significance, statistical significance, research evidence, p-values, Bayes factors, reproducibility, replicability

## Abstract

Seeking to address the lack of research reproducibility in science, including psychology and the life sciences, a pragmatic solution has been raised recently:  to use a stricter *p* < 0.005 standard for statistical significance when claiming evidence of new discoveries. Notwithstanding its potential impact, the proposal has motivated a large mass of authors to dispute it from different philosophical and methodological angles. This article reflects on the original argument and the consequent counterarguments, and concludes with a simpler and better-suited alternative that the authors of the proposal knew about and, perhaps, should have made from their Jeffresian perspective: to use a Bayes factors analysis in parallel (e.g., via JASP) in order to learn more about frequentist error statistics and about Bayesian prior and posterior beliefs without having to mix inconsistent research philosophies.

## Argument

Seeking to address the lack of research reproducibility due to the high rate of false positives in the literature, Benjamin *et al.* (2017a); Benjamin *et al.* (2017b) propose a pragmatic solution which “aligns with the training undertaken by many researchers, and might quickly achieve broad acceptance” (also Savehn, 2017): to use a stricter *p* < 0.005 standard for statistical significance when claiming evidence of new discoveries.

The proposal is subject to several constrains in its application: (1) to claims of discovery of new effects (thus, not necessarily to replication studies); (2) when using null hypothesis significance testing (arguably Fisher’s approach, perhaps even Neyman-Pearson’s, but excluding other *p*-value-generating approaches such as resampling); (3) in fields with too flexible standards (namely 5% or above); (4) when the prior odds of alternative-to-null hypothesis is in the range 1-to-5, to 1-to-40 (stricter standards are required with lower odds); (5) for researcher’s consumption (thus, not a standard for journal rejection, although “journals can help transition to the new statistical significance threshold”; also, “journals editors and funding institutions could easily enforce the proposal”, Wagenmakers, 2017; and “its implementation only requires journal editors to agree on the new threshold”, Machery, 2017); (6) while still keeping findings with probability up to 5% as suggestive (and meriting publication if so “properly labelled”); (7) despite many of the proponents believing that the proposal is nonsense, anyway (that is, it is a quick fix, not a credible one; also Ioannidis in Easwaran, 2017; Resnick, 2017; Wagenmakers, 2017; Wagenmakers & Gronau, 2017).

The main analyses supporting the proposal were the following: (a) a calibration of *p*-values to Bayes factors under certain plausible alternatives (their Figure 1, Benjamin *et al.*, 2017b, p. 2); (b) an estimated false positive rate under certain plausible prior odds of alternative-to-null hypotheses as a function of power (their Figure 2, Benjamin *et al.*, 2017b, p. 3); and (c) a “critical mass of researchers now endors[ing] this change” (a.k.a., the 72 co-authors). Meanwhile, the proposal downplays the costs due to increasing sample size in order to ensure enough research power, the potential increase in false negatives and misconduct, and the existence of alternative solutions well known by, at least, some of the authors (despite Machery’s, 2017, claim to the contrary).

**Figure 1.**
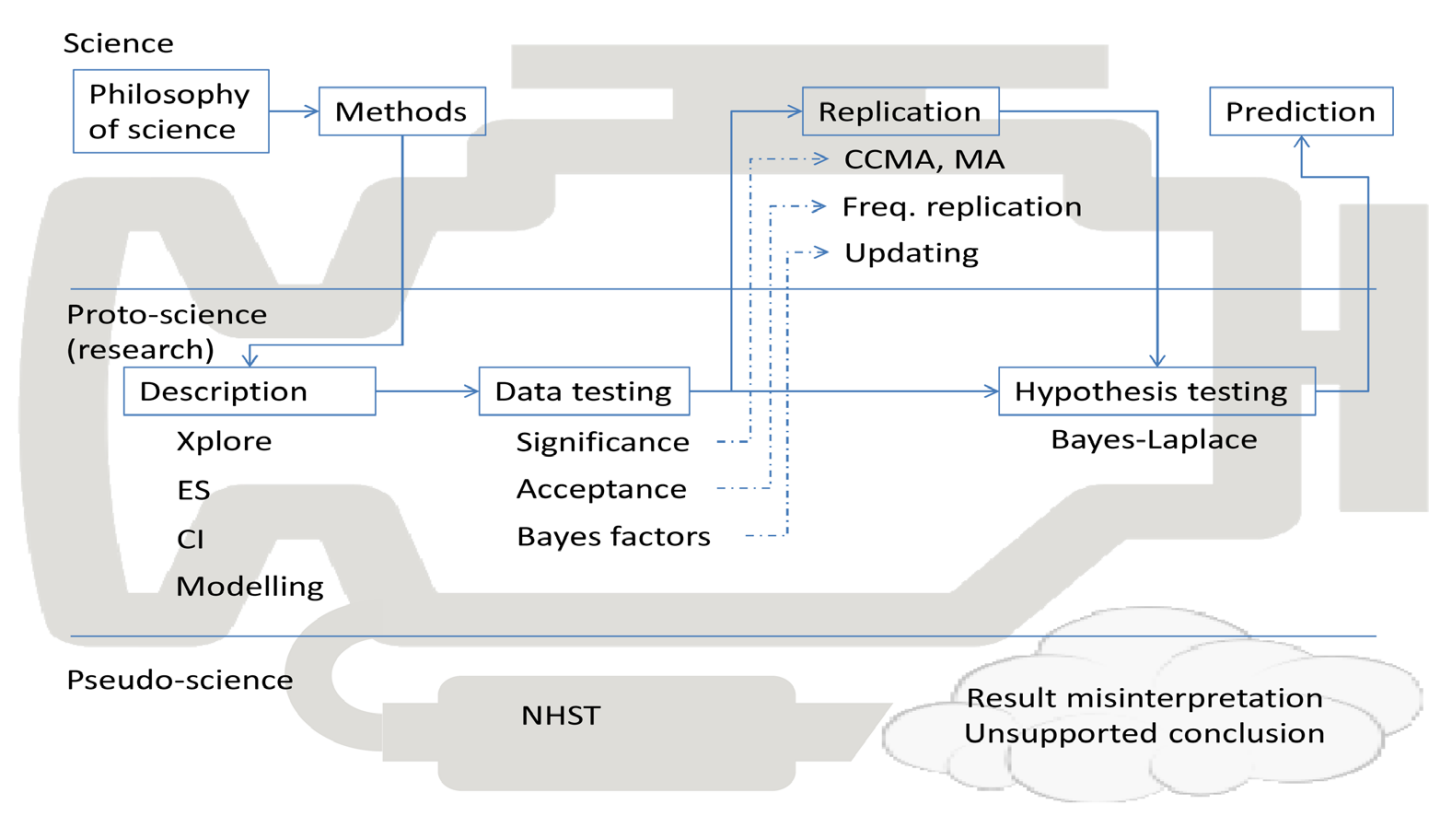
The engine of science (conceptual illustration). Xplore = exploratory data analysis; *ES* = effect size; *CI* = confidence interval, credible interval; *CCMA* = continuous cumulating meta-analysis; *MA* = meta-analysis; *Freq. replication* = frequentist replication; *NHST* = null hypothesis significance testing (as in Perezgonzalez, 2015).

Notwithstanding its potential impact, Benjamin *et al.*’s (2017a); Benjamin *et al.*’s (2017b) proposal is not recognizant of the entire engine of science ( Figure 1), which has motivated an even larger mass of authors to offer their counter-arguments.

## Counter-arguments

From **Philosophy of science**, Mayo (2017a), in particular, provides important counter-arguments against the philosophical standing of Benjamin *et al.*’s proposal. Quoting Greenland *et al.* (2016), Mayo asserts that whether *p*-values exaggerate the evidence against the null hypothesis depends on the philosophical background within which such claim is made: It may seem so from a Bayesian perspective, but both from a frequentist and an error statistics perspective, it is the Bayes factor which exaggerates the evidence (also 2017e). “I find it especially troubling”—she continues—“to spoze an error statistician…ought to use a Bayes Factor as the future gold standard for measuring his error statistical tool…even though Bayes Factors don’t control or measure error probabilities” (2017b). Furthermore, Mayo pinpoints the (old) fallacy of transposing the conditional, whereby the (error) probability of a test is confused with the (posterior) probability of a belief (also Trafimow *et al.*, 2017). And despite “60 years (sic) old…demonstrations [showing] that with reasonable tests and reasonable prior probabilities, the disparity vanishes…they still mean different things” (2017c). In particular, “in an adequate account [of severity testing], the improbability of a claim must be distinguished from its having been poorly tested. (You need to be able to say things like, ‘it’s plausible, but that’s a lousy test of it.’)” (2017d). And “the method for such checking is significance tests!” (2017b).

From **Methodology**, a large number of critics have faulted Benjamin *et al.*’s disregard of what is really affecting replication in order to focus on a relatively minor issue. Mayo, for example, lists biasing selection effects, cherry-picking, multiple testing, hunting for significance, optional stopping, violated statistical assumptions, missing or irrelevant statistical-substantive links, and questionable measurements as the main culprits in flawed findings and lack of reproducibility (2017a–e; also Gelman & McShane, 2017; Gelman, 2017b). Lakens *et al.* (2017) add lack of experimental redundancy, logical traps, research opacity, and poor accounting of sources of error, as well as the risks of reduced generalisability and research breadth were Benjamin *et al.*’s proposal to succeed. Methodological concerns were also raised by Amrhein & Greenland (2017); Black (2017); Byrd (2017); Chapman (2017); Crane (2017); Ferreira & Henderson (2017); Greenland (2017); Hamlin (2017); Kong (2017); Lew (2017); Llewelyn (2017); Martin (2017); McShane *et al.* (2017); Passin (2017); Steltenpohl (2017); Trafimow *et al.* (2017); Young (2017); Zollman (2017); and Morey (2017). Some researchers even propose the use of preregistration as a way of minimizing above problems ( Hamlin, 2017; Llewelyn, 2017; van der Zee, 2017)

The pseudoscientific **NHST element** (as conceptualized in Perezgonzalez, 2015) is the cornerstone of Benjamin *et al.*’s proposal, as it mixes Fisher’s tests of significance with Neyman-Pearson’s alternative hypotheses, Type II errors and power estimation, and with Bayesian prior probabilities, in order to argue about the false positive rate as the culprit for the lack of replicability. Indeed, the false positive rate argument has been heavily criticized by several authors: Mayo (2017b; also Byrd, 2017) chiefly derides as questionable the prior probabilities given to the hypotheses; Colquhoun (2017) denounces the credibility of Benjamin *et al.*’s false positive rate calculation for some of the prior probabilities used, which he sees as a rather liberal rate; Chapman (2017; also Byrd, 2017) claims that the prior probability ratios for the hypotheses are not realistic; Llewelyn (2017) raises an argument on prior probabilities and their combination with accumulated evidence in order to estimate the probability of future replication, not finding a more restrictive level of significance an adequate antecedent for increasing such probability; Krueger (2017) adds that, under the new proposal, the proportion of false negatives rises faster than the speed at which false positives drops, leading Phaf (2017) to wonder whether, because of it, “it makes more sense to increase, rather than reduce, the significance level for novel findings”; Kong (2017) states that the false positive rate formula is misleading and finds the expected false positive rate when testing the conventional 5% threshold.

Furthermore, Perezgonzalez (2017) argued that the misinterpretation of *p*-values as evidence in favour or against a hypothesis has more to do with the pseudoscientific use of NHST than with frequentist testing proper (also Amrhein & Greenland, 2017; Mayo, 2017b; McShane *et al.*, 2017). As Benjamin *et al.*’s proposal is made within the pseudo-philosophical argument of NHST (e.g., confusing statistical and substantive significance; Mayo, 2017c), a lower threshold of significance does not improve such ‘magical thinking’ (also Argamon, 2017; Diebold, 2017; Greenland, 2017; Krueger, 2017; Phaf, 2017). Lakens *et al.* (2017; also Morey, 2017; O’Rourke, 2017) equally warn that the proposal exaggerates the focus on single *p*-values in scientific practice, education, decision making and publication.

**Unsupported conclusions** are another consequence of pseudoscientific thinking, and McShane *et al.* (2017) and Amrhein & Greenland (2017) highlight the risks of studies published under the new banner exaggerating effect sizes and overconfidence in significant results while discounting non-significant findings. De Brigard (2017) argues, instead, for the need to be humbler and not generalize to populations, irrespective of statistical significance, effects that may only be present in the sample.

The **descriptive data analysis** element was briefly touched upon by Mayo (2017a)—who recommends abandoning significance testing in favour of inferring the magnitudes that are well (or poorly) indicated by the data (a.k.a., CIs)—and Lew (2017), Phaf (2017), and McShane *et al.* (2017)—whom argue for the need to interpret evidence in the context of other findings and theoretical models in lieu of NHST.

Regarding **data testing**, Mayo (2017a); Mayo (2017b) and Perezgonzalez (2017) have no particular problem with a lower level of significance although both reckon Benjamin *et al.*’s proposal does not address anything in particular with frequentist testing proper. Mayo equally sees such lowering unnecessary as “you might not want to be too demanding before claiming evidence of a [null] model violation” (2017b), while the “lack of replication is effectively uncovered thanks to statistical significance tests” (2017e). Young (2017) reminds us of the appropriate use of significance tests in experimental contexts. McShane *et al.* (2017) also argues the credibility of uniformly most powerful priors and tests and the loss of its connection to Bayesianism as for justifying Benjamin *et al.*’s proposal (but see Wagenmakers & Gronau’s counter-argument, 2017).

Other authors recommend continuing using *p*-values either as part of frequentist tests proper ( Bates, 2017) or without significance testing, the latter including Colquhoun (2017), Mayo (2017b; who also proposes estimating false positive rates), Greenland (2017, who proposes transforming them to bits of information against the null hypothesis), Diebold (2017); Funder (2017); Lakens *et al.* (2017, who recommend justifying whatever thresholds may be used at the design stage), McShane *et al.* (2017, who recommend using them as descriptives among other neglected factors—but see Wagenmakers & Gronau’s, 2017, counter-argument, and Gelman’s, 2017a, counter-counter-argument), and Amrhein & Greenland (2017). Argamon (2017) suggests substituting Bayesian statistics, instead.

Addressing **replication** directly, Chapman (2017) and Trafimow *et al.* (2017) point out that the problem with replication is not too many false positives but insufficient power. Krueger (2017; also McShane *et al.*, 2017, Trafimow *et al.*, 2017) chides Benjamin *et al.* for the incoherence of considering replication as order-dependent and inverting the exploratory-confirmatory nature of replication by proposing to make the former more difficult to achieve and the latter more liberal. He further chides them on whether they would disallow their own past findings at the 5% threshold. Lakens *et al.* (2017; also Crane, 2017) found biases in the analyses done by Benjamin *et al.*, and conclude that there is no [Bayesian] evidence that a lower level of significance improves replicability. They also warn of the risks of fewer availability of replication studies were the proposal to succeed (also Greenland, 2017). Gelman (2017b); Hamlin (2017); Ickes (2017); Kong (2017); Roberts (2017), and Trafimow *et al.* (2017) propose to stop the proverbial beating around the bushes and perform direct replications as a straightforward measure of replicability.

The **hypothesis testing** element (namely full Bayesian hypothesis testing) has been seldom touched upon but briefly by Llewelyn (2017), who extended the need for prior probabilities and even more accumulated evidence as pre-requisite for estimating the probability of hypotheses being ‘true’.

In conclusion, despite Benjamin *et al.*’s best intentions, their proposal only reinforces the NHST pseudoscientific approach with a stricter bright-line threshold for claiming evidence it cannot possibly claim—irrespective of how well it calibrates with Bayes factors under certain conditions—while dismissing potential consequences such as the stifling of research and its publication, an increase in false negatives, an increase in misbehaviour, and a continuation of pseudoscientific practices. Furthermore, theirs is a frequentist solution many of their Bayesian proponents do not even believe in, born out of a false belief of lacking equally simple but better-suited alternatives ( Machery, 2017).

In reality, an equally simple and better suited alternative exists, perhaps the only one the authors could be entitled to make from their Jeffresian perspective, hence our own recommendation: Use JASP, the stand-alone, free-to-download, R-based statistical software with user-friendly interface, developed by Wagenmakers’ team ( https://jasp-stats.org ^1^). JASP allows for analysing the same dataset using frequentist (Fisher’s tests of significance) and Bayesian tools (Jeffreys’s Bayes factors ^2^) without necessarily forcing a mishmash of philosophies of science (e.g., see Supplementary File 1). JASP allows for the Popperian (also Meehl’s, Mayo’s) approach to severely testing a null hypothesis but also to ascertain the credibility of tests based on the observed data. JASP allows researchers to qualitatively calibrate their significance tests to Bayes factors but also the possibility of calibrating Bayes factors to frequentist tests so as to ascertain their error probabilities. JASP is an existing simple and easy application that shows two interpretations of the same data, which aligns well with the training undertaken by frequentist and Jeffreysian researchers alike while allowing them the opportunity to learn the alternative philosophy—irrespective of which they will choose for publication—and may as well help diminish the misinterpretation of results and the reaching of unsupported conclusions. In so doing, it may even help improve replicability.

## Notes

^1^Worthy alternatives are Morey’s R package for Bayes factors ( http://bayesfactorpcl.r-forge.r-project.org), Morey’s Bayes factor calculator ( http://pcl.missouri.edu/bayesfactor), and Dienes’s Bayes factor calculator ( http://www.lifesci.sussex.ac.uk/home/Zoltan_Dienes/inference/Bayes.htm). Even better would be to skip comparing models altogether and go for full Bayesian hypothesis testing (e.g., Kruschke, 2011). Yet none of those alternatives surpass, at present, JASP’s interface and its flexibility for parallel analysis.

^2^The latest versions of JASP (e.g. 0.8.4.0; 0.8.5.1) also allows for choosing informed priors based on Cauchy, Normal, and *t*-distributions.

## Supplementary material

Supplementary file 1: **Parallel statistical analysis via JASP**. Example of data analysis using both frequentist (Fisher) and Bayesian (Jeffreys) approaches.
