# Supplementary material for: Retract p < 0.005 and propose using JASP, instead
Source: F1000Res. 2018 Feb 16;6:2122. Originally published 2017 Dec 12. [Version 2] doi: 10.12688/f1000research.13389.2 (PMC5749128; doi:10.12688/f1000research.13389.2)
Supplement: Supplementary file 1 [file f1000research-6-14775-s0000.tgz › 690356b7-1d5c-4a5a-ba85-2b940efd4400.docx]

## Appendix 1:

The following is a worked-out example using both Fisher’s and Jeffreys’s approach to data testing. The example is based on current research under examination (Vincent, 2018), albeit anonymized and adapted for this example.

In a nutshell, we used JASP to first carry out a test of significance (Fisher) in order to ascertain the probability of the data under a null hypothesis of ‘zero’ difference between independent groups. Albeit using a conventional 5% level of significance as threshold, the most this test can inform is about the probability of the data under a *t*-distribution (with appropriate degrees of freedom); therefore, whether rejecting the null hypothesis is warranted or not with the current data. For example, one of the tests returned the following results:

| **Cohen’s d** | **CI** | **t** | **df** | **p** |
| --- | --- | --- | --- | --- |
| 0.20 | [-0.53, 1.00] | 0.67 | 44 | 0.51 |

The test results are consistent throughout. A Cohen’s d = 0.2, albeit conventionally small, thus non-negligible, was not expected to be statistically significant given the low degrees of freedom (the sample size was calculated to be sensitive to a Cohen’s d = 0.5 or larger; Perezgonzalez, 2017). The confidence interval spans both sides of ‘zero’. And the *p*-value equals 0.51. Neither of these results warrant rejecting H_0_.

Unfortunately, there is little more we can learn from Fisher’s test, even when it seems obvious that the hypothesis of ‘no difference’ is plausible. We certainly cannot use Neyman-Pearson’s approach because we lack information about the alternative hypothesis (i.e., about its central limit and power).

We can, however, use Jeffreys’s approach to learn more about the plausibility of the null hypothesis, conditioned on our sample. JASP contrasts two models, both centered on ‘zero’ (thus, no need to ‘guess’ the alternative hypothesis), with the null model concentrating the entire probability spectrum on ‘zero’ (the point-null hypothesis, EJ Wagenmakers, personal communication, January 31, 2018)—which seems a reasonable model once we have learnt that no differences in effect size may be plausible—and the alternative model spreading such probability following a Cauchy distribution (akin to a *t*-distribution with one degree-of-freedom, resulting in a symmetric but rather flat distribution with heavy tails). This alternative model allows for capturing differences in effect sizes more readily than the null model. Both models thus represent an ‘extreme’ secondary test on the null hypothesis, and the resulting Bayes Factor will inform which model is more plausible.

| **BF_01_** | **CI control** | **CI experimental** |
| --- | --- | --- |
| 2.85 | [3.87, 5.00] | [3.60, 4.75] |

The results of the BF test show that the null model is almost three times more likely than the alternative model (although this would still be considered anecdotal support for such model). We can also observe that the credible intervals for both groups are quite similar both in location and spread.

Therefore, Fisher’s test helps us ascertain the error probabilities of our data (Mayo, 2010) but it is mute when results turn out to be nonsignificant, and not much helpful when they turn out otherwise (namely, because Fisher’s tests do not specify an alternative hypothesis). Jeffreys’s test allows us to learn more both about the plausibility of the null hypothesis whenever we get nonsignificant results—i.e., about how close to ‘zero’ our results are—as well as about the plausible location of the alternative hypothesis in case of significant results.

## Appendix references

Mayo D: **Error and inference.** Cambridge University Press. 2010.

Perezgonzalez JD: **Statistical sensitiveness for the behavioral sciences.** <https://osf.io/y969t>. 2017.

Vincent T: **Situational awareness of pilots in the cruis**e. Master’s thesis submitted for examination. Massey University, New Zealand. 2018.
