# Supplementary material for: Retract p < 0.005 and propose using JASP, instead
Source: F1000Res. 2018 Feb 16;6:2122. Originally published 2017 Dec 12. [Version 2] doi: 10.12688/f1000research.13389.2 (PMC5749128; doi:10.12688/f1000research.13389.2)
Supplement: Peer review history

# Peer review: Referee response for version 1

**DOI:** 10.5256/f1000research.14537.r28963

**Published:** 2018-01-12

**Authors:** Stephen Senn

**Affiliations:** 1 Competence Center for Methodology and Statistics, Luxembourg Institute Of Health, Strassen, Luxembourg; 2 School of Health and Related Research, University of Sheffield, Sheffield, UK

I consider this is a valid contribution to the debate on this topic but like many such contributions it reflects a particular viewpoint, my own contributions ^1^ in this field are no exception, and like many contributors, and, again, I am probably also guilty, there is minimal recognition that their own views are not reasonable from other perspectives. In particular (see below) they are over-fond of the adjective *pseudoscientific *and their use of it without further justification is, in my view, a form of pseudo-argumentation.

 Particular objections that I have are the following.

1. They state: "The pseudoscientific **NHST element** is the cornerstone of Benjamin *et al.*’s proposal, as it mixes Fisher’s tests of significance with Neyman-Pearson’s alternative hypotheses." This, in my opinion, is an unsubstantiated and misleading jibe. First, although this claim is often made, it is not correct that NHST is some sort of monstrous hybrid of Neyman-Pearson (N-P) and Fisherian views. It very naturally uses common elements that belong in both.(2) For instance, Fisher, although usually associated with P-values, introduced the habit of tabulating percentage points in *Statistical Methods for Research Workers*. Secondly, as Lehmann, a classic proponent of the N-P view explained in *Testing Statistical Hypotheses*, P-values are a practical way of allowing scientist who may not agree on  appropriate Type I error rates to share results efficiently. Furthermore, although I have little enthusiasm for  what Benjamin et al are doing, I think that fact that NHST seems to represent some sort of a fusion of Fisher and NP (a point that is much exaggerated and of little relevance)  is the least important aspect of what they are doing. If they had rejected everything the Fisher proposed and described what they were doing as trying to convince diehard N-P acolytes that the common 5% level was better changed to 0.5%, it would have zero technical effect on what they are proposing and would not make it either better or worse. Whatever problems or virtues there are with the Benjamin et al proposal, the Fisher, N-P fusion is a complete red herring.
2. Will JASP and Bayes Factors (BF) really save statistical inference? I doubt it. One of the problems with the Jeffreys approach is the huge influence that the lump of probability on the 'null' has on the posterior inference. NHST is far less dependent on this and, in a context in which I work, that of drug development, the practical challenge is to avoid recommending a treatment that is worse. it is a strength of NHST that one does not have to worry too much about whether one is talking about precise(or point hypotheses) on the one hand or dividing ones on the other. Of course, Bayesians could regard this as not a strength but a weakness, on the lines that if it *does* make a difference to Bayesian posterior statements ( and the difference can be enormous) it *ought* to in the NHST context. (It is a very common habit of Bayesians to abrogate the right of judging other systems by theirs.) However, I also note that amongst the Bayesian commentators on the recent ASA statement, some dismissed precise hypotheses as being completely irrelevant.
3. Like many commentators, they appear to accept much of the replication crisis at face value. However, there are four aspects here that are important. i) Gaming can effect any system (as they recognise)  ii) Much of the discussion has taken it as obvious that  failure to replicate is a problem of the original study *only*, rather than a shared problem. However, it can only be the former if the second study is perfect and that includes not being subject to sampling variation itself. What thus becomes important is to judge how well studies of infinite size are predicted, not those that just happen to be the same size as the original (2). iii) It assumes that what is important is the extent to which the P-value predicts the P-value but there is no claim under NHST that this is so. What is relevant is the extent to which it predicts the sign of the true difference Furthermore, to shackle the Bayesian approach with the same standard (what's sauce for the goose is sauce for the gander) would require that a high BF predicted a further high BF ( *using data from the subsequent study only*) with high probability. JASP on its own will not deliver this. iv) Finally, if the only replication that matters is failure to replicate 'significance' then the proposal of Benjamin et al is nowhere near radical enough. 'Significance' must never be granted. On the other hand, if false negatives are also a problem, and not just false positives, then the solution has to be rethought from the beginning. (To be fair to the authors, they do cite Krueger's, 2017 warning about this but it is unclear to me to what extent they take it on board in what they are proposing.)

However, putting aside my objections to some of the polemics of the article, the idea of supplementing P-values by other inferential statistics strikes my as being sensible and I certainly approve their suggestion of keeping Bayes factors separate from P-values. In fact, I have no objection to Bayes Factors provided that their very tentative nature is recognised and I certainly sign up to the idea of the utility of having different ways of looking at data ^3^ ^4^.

I have read this submission. I believe that I have an appropriate level of expertise to confirm that it is of an acceptable scientific standard.

## References

[1] SennS: Two cheers for P-values?. *J Epidemiol Biostat*.2001;6(2) :193-204; discussion 205 1143449910.1080/135952201753172953

[2] SennS: A comment on replication,p-values and evidence S.N.Goodman,Statistics in Medicine 1992;11:875-879. *Statistics in Medicine*.2002;21(16) : 10.1002/sim.1072 2437-2444 10.1002/sim.1072 12210627

[3] GigerenzerGMarewskiJ: Surrogate Science. *Journal of Management*.2015;41(2) : 10.1177/0149206314547522 421-440 10.1177/0149206314547522

[4] SennSJ: You may believe you are a Bayesian but you are probably wrong.Rationality, Markets and Morals.2011;48-66.

# Peer review: Referee response for version 1

**DOI:** 10.5256/f1000research.14537.r28966

**Published:** 2017-12-21

**Authors:** Juan Carro Ramos

**Affiliations:** 1 University of Salamanca, Salamanca, Spain

The authors discuss the article by Benjamin et al., 2017 and their proposal of p <.005 as a possible solution to the lack of research reproducibility due to the high rate of false positives in the literature. The article reviews the current debate topics on NHST and contributes to the reflection on how to advance towards a Science that allows accumulating valid scientific knowledge. Suggestions: Write down at the end of the article a simple example where the result is interpreted with NHST and with the Bayes factor and the utility of JASP is presented.

I have read this submission. I believe that I have an appropriate level of expertise to confirm that it is of an acceptable scientific standard.

# Response

**Published:** 2017-12-22

**Authors:** Jose Perezgonzalez

**Affiliations:** Massey University, New Zealand

Thank you very much for your prompt review. I will append an example to address your suggestion as soon as a current research using a double frequentist/Jeffresian approach is concluded.

# Peer review: Referee response for version 1

**DOI:** 10.5256/f1000research.14537.r28961

**Published:** 2017-12-14

**Authors:** Patrizio Tressoldi

**Affiliations:** 1 Department of General Psychology, University of Padova, Padova, Italy

This is a timely and well organized synthesis of the debate ignited by Benjamin *et al*. ’s, 2017 influential paper which is still going on.

 My only suggestions are the followings:

 - to add a legend to the acronyms in Figure 1, e.g. ES, CI, etc.;

 - to correct the statement that the JASP software allows only a Jeffreys’s Bayes Factor given that in the present .8.4.0 version, the user can also choose informed priors based on Cauchy, Normal or t distributions;

 - I think that a final synoptic table related to the information offered by the different statistical indices, i.e., *p* value, CI, Bayes Factor _H1/H0_, Effect Size, etc., could help the readers acknowledge the different informational value among them and consequently to choose those ones which answer better the questions they ask their data.

I have read this submission. I believe that I have an appropriate level of expertise to confirm that it is of an acceptable scientific standard.

# Response

**Published:** 2017-12-22

**Authors:** Jose Perezgonzalez

**Affiliations:** Massey University, New Zealand

Thank you very much for your prompt review. I will upload a new version incorporating two of the suggestions made (plus proper acknowledgement). I will append an example to address the third suggestion as soon as a current research using a double frequentist/Jeffresian is finished.
